# Treatment outcomes of two surgical techniques in secondary reconstruction of unilateral cleft lip and ala nasi utilizing anthropometry assessment: a randomized controlled trial

**DOI:** 10.1186/s40902-024-00456-3

**Published:** 2025-01-02

**Authors:** Ebrahim Humadi, Mawia Karkoutly, Zafin Kara Beit

**Affiliations:** 1 Department of Oral and Maxillofacial Surgery, Faculty of Dentistry, Damascus University, Damascus, Syrian Arab Republic; 2 Department of Pediatric Dentistry, Faculty of Dentistry, Damascus University, Damascus, Syrian Arab Republic

**Keywords:** Orofacial cleft, Secondary reconstruction, Rotational flap method, Pfeifer’s wave-line incision method

## Abstract

**Background:**

An orofacial cleft significantly impacts the oral health-related quality of life of children and teenagers. Secondary reconstruction is a more complex procedure due to tissue deficiency and scarring. The study aimed to evaluate the use of Pfeifer's wave-line incision method and the rotational flap method in the secondary reconstruction of unilateral lip clefts in patients with unilateral cleft lip and ala nasi aged 5–25 years utilizing anthropometry assessment.

**Methods:**

It was a double-blinded, randomized, parallel-group, active-controlled trial with two arms. Twenty-four patients were randomly divided into two groups. Group 1: Rotational flap method. Group 2: Control group, Pfeifer's wave-line incision method. The following anthropometric measurements were considered: Lb(X):En-En: The horizontal position of the center of the cupid's bow. Ch-Lt(l:r): The distance between the cheilion and the tip of the cupid’s bow. Lt-Lb(l:r): The length of the cupid’s bow. Lt(Y)(l:r): The size of the upper lip. Lt-Lt'(l:r): The height of the vermilion at the tip of the cupid's bow. Photographs were performed according to the follow-up periods: At the baseline and before surgery (t_0_). Immediately after surgery (t_1_). Two weeks after surgery (t_2_). Six months after surgery (t_3_).

**Results:**

The rotational flap method did not outperform Pfeifer’s method in the studied anthropometric measurements. In the rotational flap method group, there was a significant improvement in the mean value of Ch-Lt(l:r) from t_0_ (1.156 ± 0.206) to t_3_ (0.962 ± 0.098), and in the average value of Lt(Y)(l:r) from t_0_ (0.944 ± 0.023) to t_3_ (0.990 ± 0.011) (*p* < 0.05). In Pfeifer’s method group, the mean value of Ch-Lt(l:r) was (1.141 ± 0.158) at t_0_, and then improved to become (1.007 ± 0.084) at t_3_ (*p* < 0.05), the average value of Ch-Lt(l:r) at t_0_ was (0.942 ± 0.026), which improved to (0.991 ± 0.012) at t_3_, and the average value of Lt-Lt'(l:r) increased from t_0_ (1.308 ± 1.174) to t_3_ (1.050 ± 0.054) (*p* < 0.05).

**Conclusion:**

Pfeifer's wave-line incision and rotational flap methods achieve similar aesthetic results in the appearance of the lip or Cupid's bow after a 6-month follow-up.

**Trial registration:**

ISRCTN registry, ISRCTN36320776, registered 06 November 2024.

## Introduction

A cleft lip is a congenital abnormality that affects the orofacial region and manifests as a gap or fissure in the upper lip. It is considered one of the most common congenital abnormalities [1]. Out of every 700 natural births, one newborn has an orofacial cleft [2]. An orofacial cleft significantly impacts the oral health-related quality of life, functional well-being, and social-emotional well-being in children and teenagers [3].

Although surgeons try to achieve optimal results in primary lip reconstruction in patients with cleft lip and palate, many cases require secondary reconstruction to improve the functional and aesthetic outcomes [4]. The secondary reconstruction is a more complex procedure due to tissue deficiency and scarring resulting from complications in wound healing and lack of surgeon experience [5]. Oral and maxillofacial surgery is moving towards a new era. However, to date, secondary reconstruction has not been eliminated [6]. Choosing the optimal surgical technique and reconstruction time is one of the challenges facing the surgeon, given that the patient undergoes many surgical procedures in the early stage of life [7]. The appropriate technique aims to obtain optimal results and reduce the number of interventions. Short lip length, lack of thickness, and lip distortion, especially the vermilion mucosal layer, are the challenges facing surgeons during secondary reconstruction [8].

A rotation flap is a curved flap of skin and underlying tissue that pivots around a specific point to fill a defect. Each nasal sidewall provides rotation flaps that can be used to cover the defect by rotating them towards the midline. If the defect is not centrally located, this flap may distort the symmetry of the nose [9]. In 1970, Pfeifer introduced the wave-line incision method. The Pfeifer's incision is short, curved waves that are then brought together in a straight line, aiding in the expansion of the tissue's length and width [10, 11]. The dimensions of the cleft vary between individuals, which leads to variation and differences in the surgical procedure [10, 11]. The wave-line incision method allows for various modifications in the shape of the surgical incision to suit the variety of clefts of the lip. Therefore, surgical techniques aim to increase the lip length and thickness and to reduce the scar size [11, 12]. The study aimed to evaluate utilizing Pfeifer's wave-line incision method and the rotational flap method in the secondary reconstruction of unilateral lip clefts in patients with unilateral cleft lip and ala nasi aged 5–25 years utilizing anthropometry assessment. The null hypothesis is that Pfeifer's wave-line incision method would not outperform the rotational flap method in enhancing the facial anthropometry measurements. To the authors’ knowledge, no study has ever compared the previous two surgical methods in the secondary reconstruction of unilateral lip clefts in patients with unilateral cleft lip and ala nasi. Such trials provide surgical techniques for enhancing aesthetic and functional treatment outcomes, improving the oral health-related quality of life of unilateral cleft lip and ala nasi patients.

## Materials and methods

### Study design and ethical considerations

It was a double-blinded, randomized, parallel-group, active-controlled trial with two arms. The study took place at the Oral and Maxillofacial Surgery Hospital, Faculty of Dentistry, Damascus University, Syria, from August 2022 to March 2024. Informed consent was obtained from the participant or the participant's legal guardian under eighteen years old, and the participant's anonymity was preserved. The research was conducted in strict compliance with the CONSORT guidelines [13] and the World Medical Association Declaration of Helsinki concerning experiments with human subjects, as updated in 2013 [14]. Approval for the study was granted by the Ethics Committee of Damascus University (N4070), and the trial was authorized and listed in the ISRCTN registry (ISRCTN36320776) on 06/11/2024.

### Sample size calculation

The sample size was calculated utilizing G*Power version 3.1.9.4 (Heinrich Hein Universität Düsseldorf, Germany). A sample size of 24 patients achieved an effect size d of (1.19), 80% power (1 − β error probability), and a significance level (α error probability) of 0.05. The effect size was estimated based on a pilot study for four samples [15].

### Eligibility criteria and grouping

Inclusion criteria

1. Patients have unilateral cleft lip with/or without cleft palate.
2. Patients aged 5–25 years.
3. Patients have previously undergone primary reconstruction of unilateral cleft lip.
4. Patients have a non-aesthetic scar.
5. Patients have a deficiency in the length of the upper lip.
6. Patients have a defect in the vermilion mucosal layer of the lip [16, 17].

Exclusion criteria

1. Patients who have any systemic condition are contraindications for surgery and general anesthesia.
2. Patients have undergone corrective scar treatment [16, 17].

### Randomization and blinding

Participants were randomly divided into two groups utilizing a simple randomization method by flipping a coin. This was a double-blind trial where both participants and outcome assessors were kept unaware of group assignments and the study's purpose. However, blinding the surgeon was not feasible.

### Grouping and intervention

All participants underwent clinical and laboratory examinations. Photographs were taken according to the Frankfurt horizontal plane. Individuals referred to the Oral and Maxillofacial Surgery Hospital underwent an assessment for eligibility. Out of 31 patients, 24 patients were randomly divided into two groups:

- Group 1: Rotational flap method (*n* = 12).
- Group 2: Control group, Pfeifer's wave-line incision method (*n* = 12).

#### Group 1

**Fig. 1:**
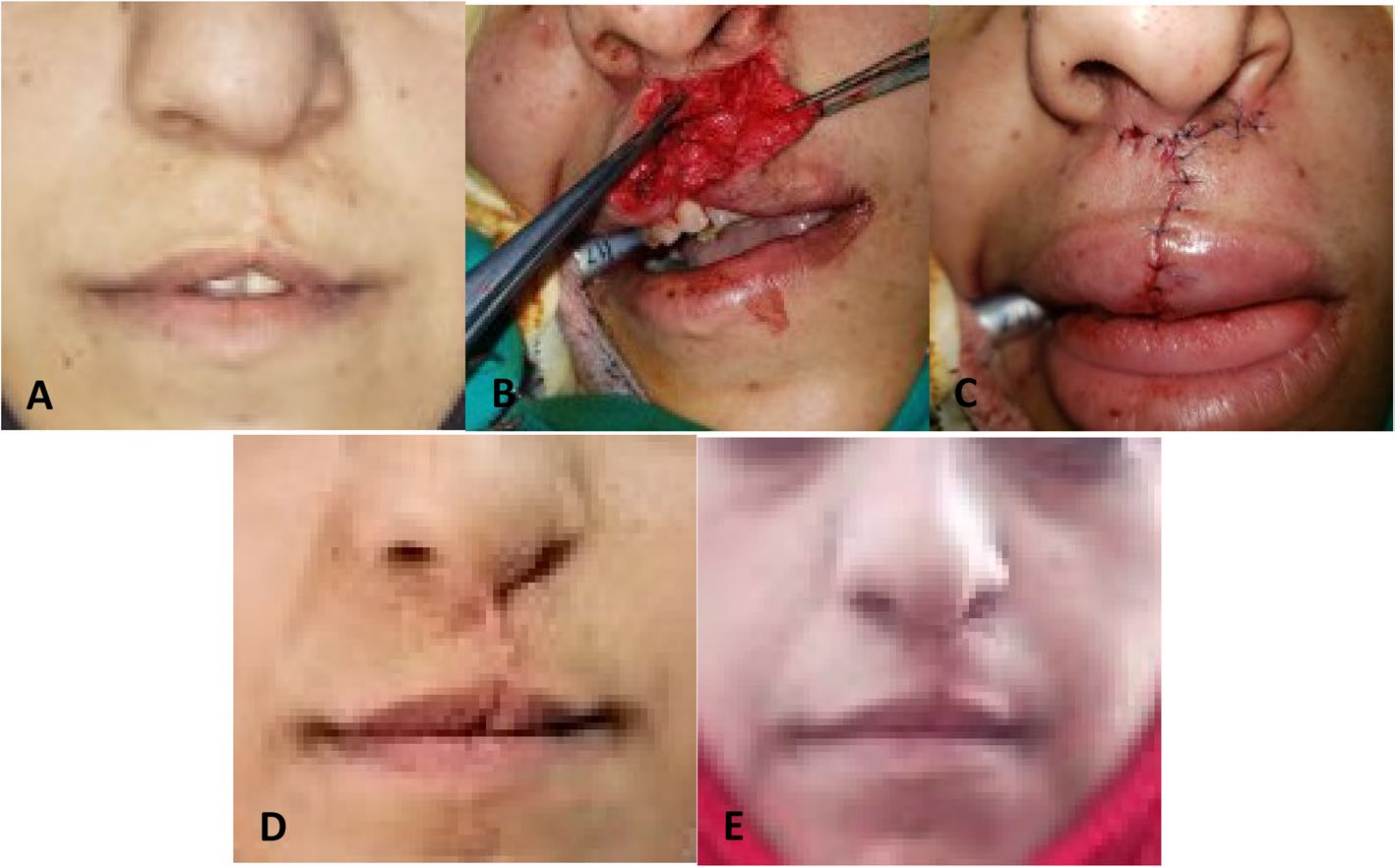
Rotational muscle group. **A** At the baseline, before surgery (t_0_). **B** During surgery. **C** Immediately after surgery (t_1_). **D** Two weeks after surgery (t_2_). **E** Six months after surgery (t_3_)

The surgical incision was made on the edges of the scar after marking the incision with methylene blue (Terry's Polychrome Methylene Blue 2% Aqueous, Polysciences Inc., Warrington, United States). The scar was then removed. The incision was bound to the muscles and the mucous membrane, after which the nasal labial muscles were dissected from the skin and mucous membrane, resulting in two muscle sections, a middle section, and a lateral section. The median part was divided into two muscle flaps, the first containing the depressor septi nasi muscle and the second containing the orbicularis oris muscle, and the lateral part into two muscle flaps, the first containing the levator labii superioris alaeque nasi muscle and the second containing the orbicularis oris muscle. Four muscle flaps were obtained. The muscle flap containing the levator labii superioris alaeque nasi muscle was sutured to the periosteum of the anterior nasal spine. The depressor septi nasi muscle flap was used to cover the previous flap and was sutured superficially. The lateral part of the orbicularis oris muscle was sutured to the periosteum of the anterior nasal spine and the levator labii superioris alaeque nasi muscle. Then, the free edges of the two orbicularis oris muscle flaps were sutured with a horizontal mattress suture to form the philtrum with a 4–0 Vicryl suture (Vicryl suture 4–0, V304H, RB-1 needle, 70 cm purple, Ethicon Inc., New Jersey, United States) (Fig. 1).

**Fig. 2:**
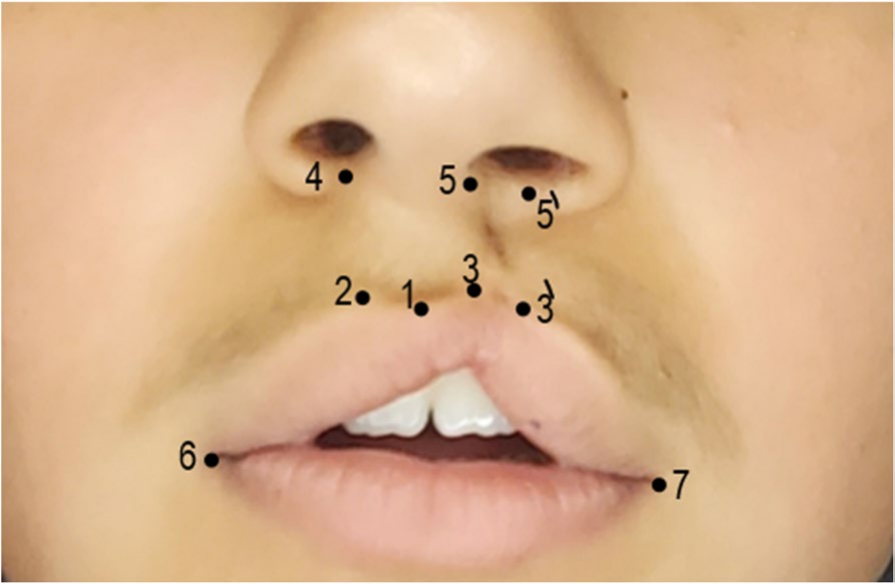
Landmarks of Pfeifer's wave-line incision method

The following landmarks were considered (Fig. 2):

- (1): The center of the upper lip at the vermilion border.
- (2): The peak of Cupid’s bow on the unaffected side.
- (3): The virtual peak of Cupid's bow on the affected side, which is a distance from point (1) equal to the distance between points (1) and (2).
- (4): A point on the nasal floor distal to the columella base by 2 mm on the unaffected side.
- (5): A point on the nasal floor located 2 mm distal to the columella base on the affected side.
- (6): The oral commissure on the unaffected side.
- (7): The oral commissure on the affected side.
- (3'): A point on Cupid's bow and the scar corresponding to the end (3) at a distance from (7) equal to the distance (2) from (6).
- (5'): A point opposite to point (5), which is the same distance from the base of the ala nasi as point (4), is from ala nasi on the unaffected side [18].

#### Group 2

**Fig. 3:**
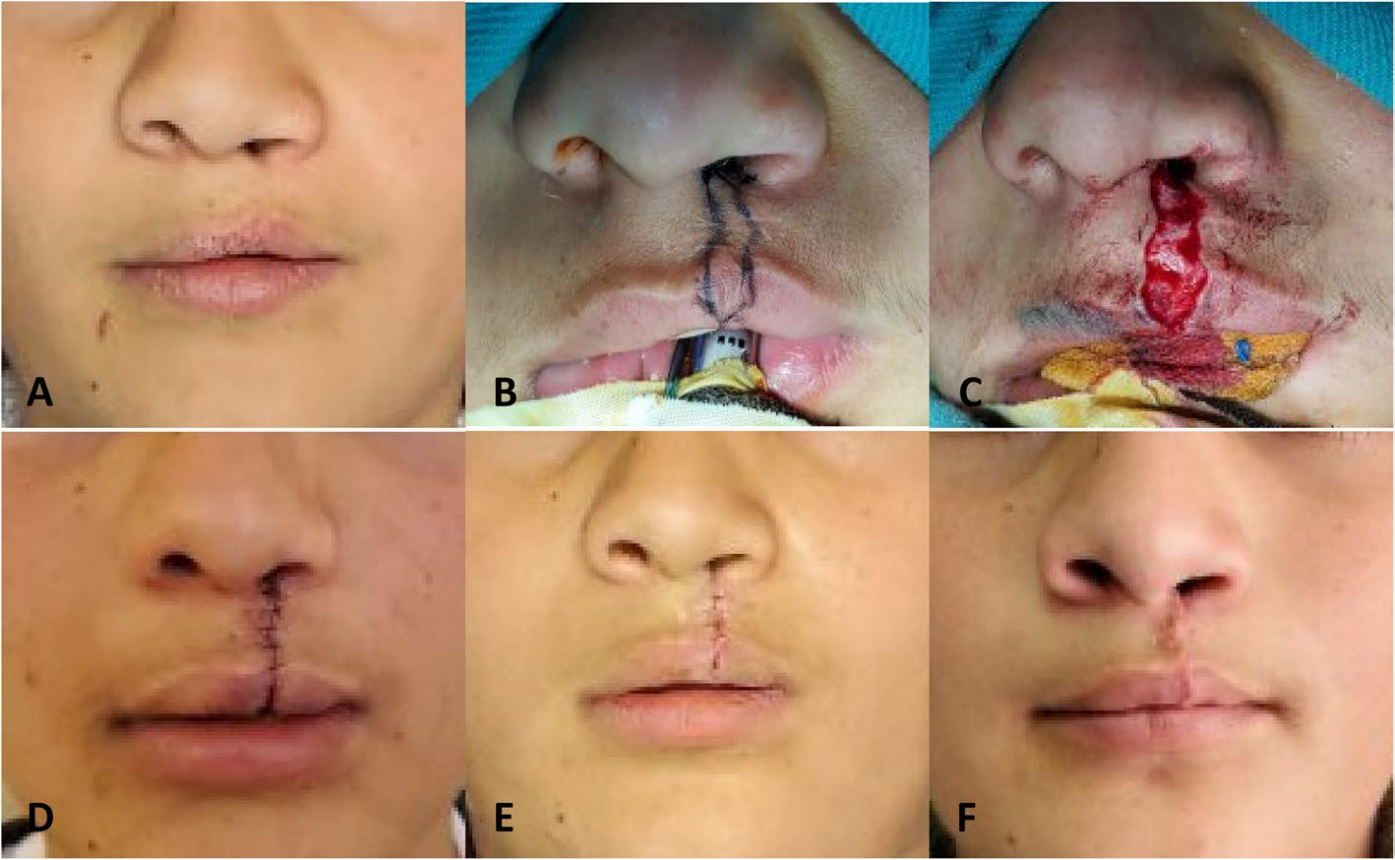
Pfeifer's wave-line incision method. **A** At the baseline, before surgery (t_0_). **B** Landmarks. **C** During surgery. **D** Immediately after surgery (t_1_). **E** Two weeks after surgery (t_2_). **F** Six months after surgery (t_3_)

The philtrum was marked utilizing a metal wire and measured from the oral commissure on the unaffected side up to the peak of Cupid's bow on the same side. The metal wire was placed on the affected side to mark the virtual peak of Cupid's bow on the affected side. To determine the number and shape of the waves, the distance (2–4) was measured using an adaptable wire, then it was adjusted in the form of waves between the two points (3–5) and (3′ 5'), and the last wave went towards the vermilion at a right angle, and the waves intersect at the nostril. Local anesthetic 2% lidocaine with epinephrine 1:80,000 solution (2% Lidocaine HCL Injection, Huons Co., Ltd, Seongnam) was injected. The incision was made with a Surgical Scalpel Blade No.11. (No.11, Swann-Morton® Ltd., Sheffield, England), reaching the muscle layer to remove the scar. The muscles were released to 4–6 mm under the skin, and several transverse incisions were performed. Suturing was initiated with a stitch guide on the vermilion and pulled down to evaluate the position of the waves. The oral mucosa was sutured from the nose towards the red lip, then the orbicularis oris muscle from the vermilion towards the nose with 4–0 Vicryl suture, then suturing subcutaneous layer with 5–0 Vicryl suture (Vicryl suture 5–0, V391H, FS-2 needle, 45 cm purple, Ethicon Inc., New Jersey, United States), and the skin with 5–0 nylon suture (Ethilon suture 5–0, 698H, P-3 needle, 45 cm black, Ethicon Inc., New Jersey, United States) (Fig. 3) [19].

### Outcome measure and follow-ups

**Fig. 4:**
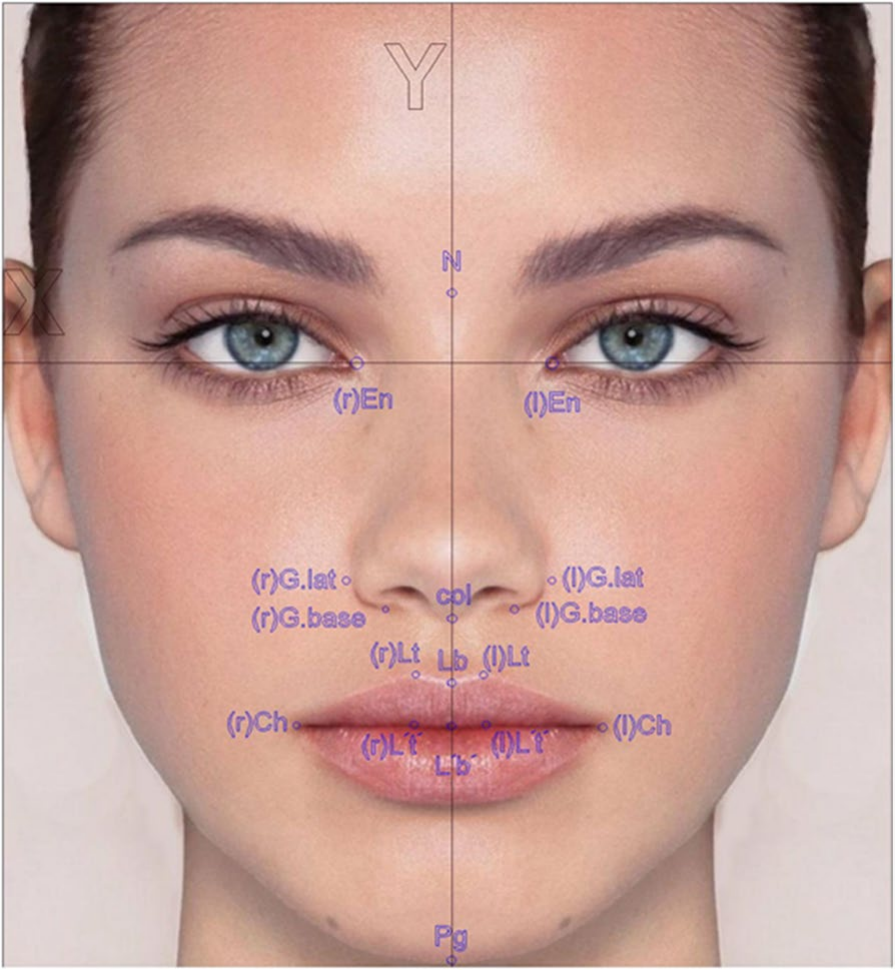
Anthropometric measurement abbreviations

The following anthropometric measurements were considered utilizing AutoCAD software (Autodesk AutoCAD 2012, Autodesk Inc., San Francisco, United States) (Table 1 and Fig. 4):

- Lb(X):En-En: The horizontal position of the center of the cupid's bow.
- Ch-Lt(l:r): The distance between the cheilion and the tip of the cupid’s bow.
- Lt-Lb(l:r): The length of the cupid’s bow.
- Lt(Y)(l:r): The length of the upper lip.
- Lt-Lt'(l:r): The height of the vermilion at the tip of the cupid's bow [20].

**Table 1:** Description of anthropometric measurement abbreviations

Abbreviation	Description
En	Endocanthion
Lb	Base of the cupid's bow
Ch	Cheilion
Lt	Tip of the cupid's bow
L't'	The point corresponding to Lt at the bottom of the vermilion
En-En	The distance between endocanthions
Lb(X)	The distance between Lb and y-axis
(r)Ch-Lt	The distance between Ch and Lt on the unaffected side
(l)Ch-Lt	The distance between Ch and Lt on the affected side
(r)Lt-Lb	The length of the cupid’s bow on the unaffected side
(l)Lt-Lb	The length of the cupid’s bow on the affected side
(r)Lt(Y)	The distance between Lt and y-axis on the unaffected side
(l)Lt(Y)	The distance between Lt and y-axis on the affected side
(r)Lt- L't'	The height of the vermilion on the unaffected side
(l)Lt-L't'	The height of the vermilion on the affected side
Lb(X):En-En	Evaluating the horizontal position of the center of the cupid's bow
Ch-Lt(l:r)	Evaluating the distance between the cheilion and the tip of the cupid’s bow
Lt-Lb(l:r)	Evaluating the length of the cupid’s bow
Lt(Y)(l:r)	Evaluating the length of the upper lip
Lt-Lt'(l:r)	Evaluating the height of vermilion at the tip of the cupid's bow

The interpretation of anthropometric measurement values is presented in Table 2. Photographs were performed at successive intervals according to the follow-up periods:

- At the baseline, before surgery (t_0_).
- Immediately after surgery (t_1_).
- Two weeks after surgery (t_2_).
- Six months after surgery (t_3_).

**Table 2:** Interpretation of anthropometric measurement values

Measurement	Normal value	Value interpretation
Lb(X):En-En	0	> 0 = Horizontal displacement of the center of the cupid's bow towards the affected side
		< 0 = Horizontal displacement of the center of the cupid's bow towards the unaffected side
Ch-Lt(l:r)	1	> 1 = Asymmetry. The distance between the cheilion and the tip of the cupid’s bow is greater on the affected side
		< 1 = Asymmetry. The distance between the cheilion and the tip of the cupid’s bow is smaller on the affected side
Lt-Lb(l:r)	1	> 1 = Asymmetry in the cupid’s bow. The length of the cupid’s bow is greater on the affected side
		< 1 = Asymmetry in the cupid’s bow. The length of the cupid’s bow is smaller on the affected side
Lt(Y)(l:r)	1	> 1 = Increase in the upper lip length. The tip of the cupid’s bow is lower on the affected side
		< 1 = Decrease in the upper lip length. The tip of the cupid’s bow is higher on the affected side
Lt-Lt'(l:r)	1	> 1 = Asymmetry the vermilion. The height of the vermilion is greater on the affected side
		< 1 = Asymmetry the vermilion. The height of the vermilion is greater on the affected side

### Statistical analysis

Data analysis was conducted using IBM SPSS software version 24 (IBM SPSS Statistics® version 24, IBM Corp., New York, USA). Participants’ characteristics were summarized with descriptive statistics, shown as frequency, percentage, mean, and standard deviation (SD). The anthropometric measurements were also presented using descriptive statistics, including mean, SD, minimum (Min), and maximum (Max). The Kolmogorov–Smirnov test was used to assess the normality of the data. An Independent sample t-test was utilized to compare unpaired data, while a paired sample t-test was used for paired data. The significance level was set at 0.05 (*p* < 0.05).

## Results

**Fig. 5:**
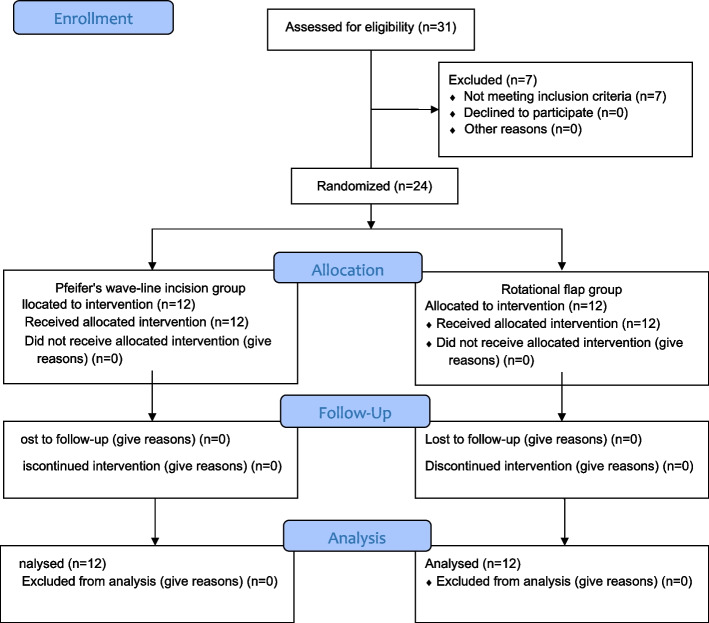
CONSORT flow diagram

The CONSORT flow diagram is shown in Fig. 5. Out of 31 patients, 24 patients were included. The mean age was (12.8 ± 5.5; range: 5–25), and around half (54.17%) of the participants were male (Table 3).

**Table 3:** Demographic characteristics of participants

Groups	n	Male		Female		Age	
		**n**	**%**	**n**	**%**	**Mean ± SD**	**Min–max**
Rotational muscle method	12	4	16.67	8	33.33	12.8 ± 4.4	9–23
Pfeifer’s group	12	9	37.50	3	12.50	12.8 ± 6.8	5–25
Total	24	13	54.17	11	45.83	12.8 ± 5.5	5–25

### Lb(X):En-En measurement

The rotational flap method did not outperform Pfeifer’s method in improving the horizontal position of the center of the cupid's bow (Table 4), and no significant change was noted between different follow-up intervals in either group (Table 5). However, in Pfeifer’s method group, the mean Lb(X):En-En value was (-0.062 ± 0.164) at t_0_ and decreased to (-0.010 ± 0.085) at t_3_ (*p* = 0.287) but in rotational flap method group, the mean value increased from (0.013 ± 0.136) at t_0_ to (0.020 ± 0.086) at t_3_ (*p* = 0.897) (Tables 4 and 5), indicating that Pfeifer’s method exhibited better outcome.

**Table 4:** Descriptive statistics and the results of independent sample t-test for Lb(X):En-En measurement

Time point	Groups	Mean ± SD	Min	Max	Mean difference	t-value	*p*-value
t_0_	Rotational muscle method	0.013 ± 0.136	-0.20	0.20	0.076	1.063	0.303
	Pfeifer’s method	-0.062 ± 0.164	-0.36	0.17			
t_1_	Rotational muscle method	0.020 ± 0.091	-0.12	0.14	0.009	0.184	0.856
	Pfeifer’s method	0.011 ± 0.115	-0.21	0.15			
t_2_	Rotational muscle method	0.026 ± 0.092	-0.09	0.18	-0.079	-0.716	0.484
	Pfeifer’s method	0.105 ± 0.319	-0.19	0.89			
t_3_	Rotational muscle method	0.020 ± 0.086	-0.13	0.15	0.030	0.744	0.468
	Pfeifer’s method	-0.010 ± 0.085	-0.15	0.11

**Table 5:** The results of paired sample t-test for Lb(X):En-En measurement

Groups	Time points	Mean difference	t-value	*p*-value
Rotational muscle method	t_0_ vs. t_1_	0.007	0.111	0.915
	t_0_ vs. t_2_	0.012	0.228	0.826
	t_0_ vs. t_3_	0.007	0.133	0.897
	t_1_ vs. t_2_	0.006	0.282	0.785
	t_1_ vs. t_3_	0.000	-0.004	0.997
	t_2_ vs. t_3_	-0.006	-0.488	0.638
Pfeifer’s method	t_0_ vs. t_1_	0.073	1.613	0.145
	t_0_ vs. t_2_	0.167	1.624	0.143
	t_0_ vs. t_3_	0.052	1.140	0.287
	t_1_ vs. t_2_	0.094	0.906	0.391
	t_1_ vs. t_3_	-0.021	-0.543	0.602
	t_2_ vs. t_3_	-0.115	-1.181	0.271

### Ch-Lt(l:r) measurement

Both methods were equally effective in improving the distance between the cheilion and the tip of the cupid’s bow (Table 6). In the rotational flap method group, there was a significant improvement in the mean value of Ch-Lt(l:r) from t_0_ (1.156 ± 0.206) to t_3_ (0.962 ± 0.098) (*p* < 0.05) (Tables 6 and 7). Similarly, in Pfeifer’s method group, the mean value was (1.141 ± 0.158) at t0, and then improved to become (1.007 ± 0.084) at t3 (*p* < 0.05) Tables 6 and 7).

**Table 6:** Descriptive statistics and the results of independent sample t-test for Ch-Lt(l:r) measurement

Time point	Groups	Mean ± SD	Min	Max	Mean difference	t-value	*p*-value
t_0_	Rotational muscle method	1.156 ± 0.206	0.776	1.510	0.015	0.173	0.865
	Pfeifer’s method	1.141 ± 0.158	0.964	1.406			
t_1_	Rotational muscle method	1.010 ± 0.109	0.837	1.180	0.057	1.216	0.242
	Pfeifer’s method	0.953 ± 0.088	0.812	1.074			
t_2_	Rotational muscle method	0.884 ± 0.300	0.125	1.173	-0.112	-1.081	0.296
	Pfeifer’s method	0.997 ± 0.085	0.895	1.115			
t_3_	Rotational muscle method	0.962 ± 0.098	0.851	1.146	-0.045	-1.049	0.310
	Pfeifer’s method	1.007 ± 0.084	0.899	1.146

**Table 7:** The results of paired sample t-test for Ch-Lt(l:r) measurement

Groups	Time points	Mean difference	t-value	*p*-value
Rotational muscle method	t_0_ vs. t_1_	-0.145	-2.730	0.026*
	t_0_ vs. t_2_	-0.271	-1.808	0.108
	t_0_ vs. t_3_	-0.194	-2.394	0.044*
	t_1_ vs. t_2_	-0.126	-1.034	0.331
	t_1_ vs. t_3_	-0.049	-1.138	0.288
	t_2_ vs. t_3_	0.077	0.773	0.462
Pfeifer’s method	t_0_ vs. t_1_	-0.187	-2.771	0.024*
	t_0_ vs. t_2_	-0.144	-2.470	0.039*
	t_0_ vs. t_3_	-0.134	-2.718	0.026*
	t_1_ vs. t_2_	0.043	2.417	0.042*
	t_1_ vs. t_3_	0.053	1.645	0.139
	t_2_ vs. t_3_	0.010	0.529	0.611

^*^Statistically significant difference between the groups (*p* < 0.05)

### Lt-Lb(l:r) measurement

The rotational flap technique did not achieve better results than Pfeifer’s approach in enhancing the length of the cupid’s bow (Table 8), and no significant differences were observed between various follow-up times in either group (Table 9). Within the rotational flap technique group, the average Lt-Lb(l:r) value rose from (0.946 ± 0.351) at t_0_ to (1.035 ± 0.152) at t_3_ (*p* = 0.396) (Tables 8 and 9). In the group utilizing Pfeifer’s method, the mean value was (1.195 ± 0.290) at t_0_, which decreased to (1.002 ± 0.065) at t_3_ (*p* = 0.116) (Tables 8 and 9).

**Table 8:** Descriptive statistics and the results of independent sample t-test for Lt-Lb(l:r) measurement

Time point	Groups	Mean ± SD	Min	Max	Mean difference	t-value	*p*-value
t_0_	Rotational muscle method	0.946 ± 0.351	0.478	1.500	-0.250	-1.644	0.120
	Pfeifer’s method	1.195 ± 0.290	0.875	1.582			
t_1_	Rotational muscle method	1.057 ± 0.140	0.877	1.306	0.048	0.806	0.432
	Pfeifer’s method	1.009 ± 0.110	0.887	1.203			
t_2_	Rotational muscle method	1.006 ± 0.133	0.801	1.211	0.042	0.706	0.491
	Pfeifer’s method	0.964 ± 0.120	0.750	1.122			
t_3_	Rotational muscle method	1.035 ± 0.152	0.772	1.214	0.033	0.596	0.560
	Pfeifer’s method	1.002 ± 0.065	0.880	1.083

**Table 9:** The results of paired sample t-test for Lt-Lb(l:r) measurement

Groups	Time points	Mean difference	t-value	*p*-value
Rotational muscle method	t_0_ vs. t_1_	0.111	0.963	0.364
	t_0_ vs. t_2_	0.060	0.590	0.572
	t_0_ vs. t_3_	0.089	0.897	0.396
	t_1_ vs. t_2_	-0.051	-0.868	0.410
	t_1_ vs. t_3_	-0.022	-0.343	0.741
	t_2_ vs. t_3_	0.029	1.571	0.155
Pfeifer’s method	t_0_ vs. t_1_	-0.187	-1.699	0.128
	t_0_ vs. t_2_	-0.232	-2.075	0.072
	t_0_ vs. t_3_	-0.194	-1.761	0.116
	t_1_ vs. t_2_	-0.045	-0.794	0.450
	t_1_ vs. t_3_	-0.007	-0.184	0.859
	t_2_ vs. t_3_	0.038	1.277	0.237

### Lt(Y)(l:r) measurement

Both techniques demonstrated equivalent effectiveness in enhancing the upper lip length (Table 10). In the group utilizing the rotational flap method, there was a significant enhancement in the average value of Lt(Y)(l:r) from t0 (0.944 ± 0.023) to t3 (0.990 ± 0.011) (*p* < 0.05) (Tables 10 and 11). Likewise, in the Pfeifer’s method group, the average value at t0 was (0.942 ± 0.026), which improved to (0.991 ± 0.012) at t3 (*p* < 0.05) (Tables 10 and 11).

**Table 10:** Descriptive statistics and the results of independent sample t-test for Lt(Y)(l:r) measurement

Time point	Groups	Mean ± SD	Min	Max	Mean difference	t-value	*p*-value
t_0_	Rotational muscle method	0.944 ± 0.023	0.919	0.981	0.002	0.182	0.858
	Pfeifer’s method	0.942 ± 0.026	0.907	0.976			
t_1_	Rotational muscle method	1.004 ± 0.014	0.983	1.026	-0.015	-0.581	0.569
	Pfeifer’s method	1.019 ± 0.078	0.978	1.225			
t_2_	Rotational muscle method	0.989 ± 0.014	0.966	1.010	-0.006	-0.311	0.760
	Pfeifer’s method	0.995 ± 0.060	0.876	1.112			
t_3_	Rotational muscle method	0.990 ± 0.011	0.973	1.005	-0.001	-0.187	0.854
	Pfeifer’s method	0.991 ± 0.012	0.973	1.005

**Table 11:** The results of paired sample t-test for Lt(Y)(l:r) measurement

Groups	Time points	Mean difference	t-value	*p*-value
Rotational muscle method	t_0_ vs. t_1_	0.060	10.363	< 0.001*
	t_0_ vs. t_2_	0.045	5.742	< 0.001*
	t_0_ vs. t_3_	0.046	5.167	0.001*
	t_1_ vs. t_2_	-0.015	-2.005	0.080
	t_1_ vs. t_3_	-0.014	-2.014	0.079
	t_2_ vs. t_3_	0.001	0.241	0.816
Pfeifer’s method	t_0_ vs. t_1_	0.077	3.007	0.017*
	t_0_ vs. t_2_	0.053	2.964	0.018*
	t_0_ vs. t_3_	0.049	6.264	< 0.001*
	t_1_ vs. t_2_	-0.024	-1.451	0.185
	t_1_ vs. t_3_	-0.028	-1.164	0.278
	t_2_ vs. t_3_	-0.004	-0.236	0.820

^*^Statistically significant difference between the groups (*p* < 0.05)

### Lt-Lt'(l:r) measurement

Both methods showed similar effectiveness in increasing the height of the vermilion at the cupid's bow tip (Table 12). In the rotational flap technique, the average value at t_0_ was (1.326 ± 0.408), which improved to (1.065 ± 0.124) at t_3_. However, this change was not statistically significant (*p* = 0.104) (Tables 12 and 13). In contrast, the Pfeifer’s method group exhibited a significant improvement in the average value of Lt-Lt'(l:r) from t_0_ (1.308 ± 1.174) to t_3_ (1.050 ± 0.054) (*p* < 0.05) (Tables 12 and 13).

**Table 12:** Descriptive statistics and the results of independent sample t-test for Lt-Lt'(l:r) measurement

Time point	Groups	Mean ± SD	Min	Max	Mean difference	t-value	*p*-value
t_0_	Rotational muscle method	1.326 ± 0.408	0.687	2.091	0.017	0.117	0.908
	Pfeifer’s method	1.308 ± 1.174	1.015	1.629			
t_1_	Rotational muscle method	1.051 ± 0.111	0.845	1.226	-0.037	-0.758	0.460
	Pfeifer’s method	1.088 ± 0.095	0.949	1.229			
t_2_	Rotational muscle method	1.068 ± 0.107	0.904	1.256	-0.030	-0.616	0.547
	Pfeifer’s method	1.098 ± 0.099	0.970	1.245			
t_3_	Rotational muscle method	1.065 ± 0.124	0.927	1.301	0.015	0.326	0.748
	Pfeifer’s method	1.050 ± 0.054	0.985	1.135

**Table 13:** The results of paired sample t-test for Lt-Lt'(l:r) measurement

Groups	Time points	Mean difference	t-value	*p*-value
Rotational muscle method	t_0_ vs. t_1_	-0.275	-1.883	0.096
	t_0_ vs. t_2_	-0.258	-1.691	0.129
	t_0_ vs. t_3_	-0.261	-1.834	0.104
	t_1_ vs. t_2_	0.017	0.713	0.496
	t_1_ vs. t_3_	0.014	0.477	0.646
	t_2_ vs. t_3_	-0.003	-0.111	0.914
Pfeifer’s method	t_0_ vs. t_1_	-0.221	-3.465	0.009*
	t_0_ vs. t_2_	-0.211	-3.847	0.005*
	t_0_ vs. t_3_	-0.258	-4.914	0.001*
	t_1_ vs. t_2_	0.010	0.782	0.457
	t_1_ vs. t_3_	-0.038	-0.998	0.347
	t_2_ vs. t_3_	-0.047	-1.324	0.222

^*^Statistically significant difference between the groups (*p* < 0.05)

## Discussion

Surgical reconstruction of secondary cleft lip deformities requires a multidisciplinary approach that includes comprehensive evaluation and planning to enhance treatment outcomes [7]. Achieving optimal results or as close to normal as possible remains a real challenge for surgeons [4]. Performing secondary reconstruction is more complex than primary reconstruction in several aspects at the surgical and psychological levels [4, 5]. Furthermore, secondary reconstruction poses challenges because of the scarring and tissue loss associated with the repaired cleft lip [4, 5]. Numerous techniques for secondary correction of cleft lips have been documented, yet a universally accepted treatment has not been established [21]. This study aimed to evaluate two surgical methods for performing secondary cosmetic repair for patients with a unilateral cleft lip and ala nasi aged 5–25 years. Patients underwent primary repair of the cleft with deformities. These two methods are Pfeifer's wave-line incision and the rotational flap methods.

The current study utilized the rotational flap method because it is more effective in correcting vermilion defects and controlling their length than other methods, such as taking grafts from the lower lip or tongue to compensate for the deficiency or lengthening the upper lip or labial tubercle deformity [22]. One frequently utilized technique is the lip-switch flap, initially introduced by Abbe et al. [23], which has undergone various modifications by different surgeons. In these methods, the vermilion, mucosa, orbicularis, and skin of the lower lip are relocated to the upper lip on a pedicle that is eventually incised. All these adaptations of the lip-switch procedure involve utilizing the entire skin of the lower lip, resulting in significant unattractive scarring. Furthermore, this method introduces abnormal tissue into the philtrum, thereby altering the appearance of the upper lip. The modified cross-lip flap, as described by Sheckter et al. [24], which focuses solely on the mucosa, vermilion, and orbicularis, is a viable option that offers excellent reconstruction while preserving additional tissue. The Pfeiffer and muscle flap rotation methods depend on the adaptability of the muscle and skin tissue to elongate the lip, contrasting with many other approaches that rely on displaced or rotated flaps, which can create unsightly scar lines. The two techniques rely on tissue adaptability, which allows for the lengthening and repairing of the lip without generating unsightly scar lines, consistent with the Reddy et al. [11] study.

Anthropometric analysis plays a pivotal role in evaluating the morphological results of facial symmetry. Bilwatsch et al. [25] emphasized the need for accurate assessment of facial features using quantitative and standard anthropometric analysis methods, with statistical analysis of the extracted results. Ideally, Lb(X):En-En should be zero because Cupid's bow center aligns with the midline. However, the average value suggests that Pfeifer’s method approaches the midline. Nevertheless, no significant statistical difference was found between the two surgical techniques, indicating that both approaches were similarly effective in addressing the cosmetic concern. Ch-Lt(l:r) indicates the symmetry present between the two halves of the upper lip. The optimal value should be 1. The present study notes a noticeable enhancement during the follow-up periods, although no statistical difference was observed between the two groups. Similarly, both methods enhance Lt-Lb(l:r), Lt(Y)(l:r), and Lt-Lt'(l:r) values, which should be equal to 1. Thus, both surgical techniques lead to noticeable aesthetic differences in the long-term appearance of the lip or Cupid's bow. The current study agreed with Adetayo et al. [26] findings, which suggested that the Tennison-Randall technique does not outperform the Millard rotation-advancement technique in enhancing the anthropometric measurements of unilateral cleft lip patients. However, Alkebsi et al. [27] suggested that the modified rotation-advancement method shows superior results compared to the conventional rotation-advancement technique in enhancing the symmetry of the Cupid's bow and effectively correcting lip height while maintaining lip width in patients with unilateral cleft. Additionally, the modified rotation-advancement approach led to improved aesthetic nasal results. The current study proposed a method for secondary reconstruction at the combined cutaneous and muscular levels of the cleft lip and nasal alar. Similarly, Ma et al. [28] outlined the layered muscle flap approach, which incorporates the levator labii superioris alaeque nasi muscle alongside the orbicularis oris muscle, as a successful technique for aesthetic reconstruction of the Cupid’s bow and vermilion in cases of secondary cleft lip repair. This method produces positive outcomes because techniques utilizing skin and mucosa flaps carry certain risks of scarring. In addition, Bagatin et al. [29] indicated that the Abbe flap technique provides favorable outcomes in the secondary reconstruction of inadequate upper lips in individuals with bilateral cleft lips. The Abbe flap is a complete-thickness composite flap that involves relocating the skin, muscle, and mucosa from the central portion of the lower lip to the upper lip. Fan et al. [30] categorized muscle reconstruction into three components focused on the nasal floor, white lip, and vermilion to fix the orbicularis oris for the correction of deformities resulting from secondary cleft lip. The three-unit muscle repair technique has proven to be effective and practical for secondary repair. This comprehensive approach can lead to better aesthetic and functional outcomes. Jung et al. [31] introduced a rotation-advancement technique for the reconstruction of perioral muscles and lengthening of the lip in cases of complete unilateral cleft lip without skin measurement. The technique employed allowed for the elongation of the lip and achieved symmetry during the correction of a complete unilateral cleft lip.

The main limitation of the study is that the majority of the patients underwent surgery before the end of the growth period. Thus, the final evaluation of the results cannot be made until the end of facial growth. In addition to the short follow-up periods. However, the earlier the intervention, the better the surgical scar [12]. In addition, blinding the surgeon was not feasible. Because of the different physical components of the intervention, blinding surgeons in surgery trials is not always possible [32]. This would introduce potential bias in the execution of surgical techniques. Furthermore, the six-month follow-up period is relatively short, and conducting the study at a single center limits its external validity. Thus, it is recommended to carry out the trial at a multi-center level with a longer follow-up period to bolster the generalizability of the findings and strengthen the study's conclusions [33]. In addition, incorporating additional qualitative evaluations, such as patient satisfaction and scar visibility assessments, would enrich the anthropometric data and provide a more comprehensive evaluation of outcomes.

## Conclusions

Using either Pfeifer's wave-line incision or rotational flap methods achieves similar aesthetic results in the appearance of the lip or Cupid's bow after a 6-month follow-up in secondary reconstruction of unilateral cleft lip and ala nasi. In addition, the slight differences after surgery are often invisible or statistically insignificant.

## Data Availability

The datasets used and/or analysed during the current study are available from the corresponding author on reasonable request.

## Abbreviations

Lb(X):En-En: The horizontal position of the center of the cupid's bow.
Ch-Lt(l:r): The distance between the cheilion and the tip of the cupid’s bow.
Lt-Lb(l:r): The length of the cupid’s bow.
Lt(Y)(l:r): The length of the upper lip.
Lt-Lt'(l:r): The height of the vermilion at the tip of the cupid's bow
